# Epidemiologic Profile of Overweight and Obesity in Abidjan, Ivory Coast: A Cross-Sectional Study

**DOI:** 10.5334/aogh.2755

**Published:** 2020-04-29

**Authors:** Stephane Parfait Sable, Kaina Yan, Apollinaire Yapi, Denise Djokou Kpebo, Kokora Franck Ekou, Puriffine Odile Sassor Ake-Tano, Orsot Ekissi Tetchi, Eugene Yao Konan, Dinard Kouassi, Chengsong Wan

**Affiliations:** 1 Nutrition Unit of National Institute of Public Health, CI; 2 Felix Houphouet Boigny University, CI; 3 Department of Microbiology, School of Public Health, Southern Medical University, Guangzhou, CN; 4 Key Laboratory of Tropical Disease Research of Guangdong Province, Southern Medical University, Guangzhou, CN; 5 Health System Unit of National Institute of Public Health, CI

## Abstract

**Background:**

In sub-Saharan Africa, the prevalence of overweight and obesity is high, and it is estimated to increase within the next ten years. In Ivory Coast, the rise in and public health consequences of overweight and obese people are evident. Moreover, data concerning this status are scarce, old, local, and describe only a small sample of the population.

**Objective:**

This study has been conducted in order to describe the epidemiologic profile of overweight and obese people in Ivory Coast and identify the potential risk factors of obesity.

**Methods:**

From January 2014 to July 2017, 2,643 patients aged 17–70 years old from Abidjan of Ivory Coast were recruited. Statistical analysis was carried out using SPSS 20.0. Chi-square test and binary logistic regression analysis were used to identify risk factors for overweight and obesity.

**Results:**

Most of our patients were females (86.3%) with an estimated average age of 43.7 ± 12.19 years. Among 2,643 patients recruited in this study, 83.3% were obese and 87.2% were affected by central abdominal obesity. Binary logistic regression analysis identified seven factors significantly associated with overweight and obesity, including females (OR: 2.06; 95% CI [1.58–2.68]), >54 years old of age (OR: 3.71; 95% CI [1.84–7.50]), occupation of salesperson and traders (OR: 2.42; 95% CI [1.78–3.29]), ethnic group of North Mande ethnicity (OR: 1.47; 95% CI [1.07–2.02]), family history of obesity (OR: 1.96; 95% CI [1.46–2.63]), ≥150 minutes of sport practice (OR:0.72; 95% CI [0.55–0.96]), and parous females (OR: 1.63; 95% CI [1.11–2.38]).

**Conclusions:**

Overall, gender (female), older age, and occupation were associated with greater risks of overweight and obesity in patients. Ethnic group, pregnancy and family history of obesity posed a lower but significant risk for obesity. More sport practice played a protective role against the acquisition of overweight and obesity.

## Introduction

Described as a global epidemic, overweight and obesity could soon overwhelm both developed and developing countries [1,2]. In 2014, 39% of adults were overweight, and 11% of men and 15% of women were obese [3]. In sub-Saharan Africa, where approximately 35% of adult women are either overweight or obese, overweight and obesity are estimated to increase by about 35% within the next 10 years at a rate of about 25% per year [4,5]. The reasons for this rapid growth include globalization, increase in wealth and urbanization that leads to changes in food supply systems, changes in diets, declining levels of physical activity, and changes in the gut microbiome [6].

Furthermore, the health and economic implications of overweight or obesity are severe, since they constitute a major risk factor for many non-communicable diseases (NCDs) like arthritis, cancers, diabetes mellitus, hypertension, and cardiovascular diseases, which were leading major causes of death in 2012 [7,8]. In 2010, overweight and obesity were estimated to cause 3.4 million deaths, 3.9% of years of life loss, and 3.8% of disability-adjusted life years (DALYs) globally [9]. Peeters et al. concluded that obesity in adulthood is associated with a decrease in life expectancy of about seven years, in both men and women, and is a powerful predictor of death at older ages [10]. Estimates of non-communicable disease-related mortality in Africa indicate that there were approximately 2.1 million deaths in 2010, up by 46% from 1990 and most are associated with obesity [11]. Current projections indicate that the largest increase in non-communicable disease-related deaths will occur in Africa by 2020 and by 2030, and these deaths are projected to exceed the combined deaths from communicable and nutritional diseases, and maternal and perinatal deaths [7]. Concerning the economic implications, the costs generated by obesity and its complications are very high and are estimated to increase in studies focusing on European countries, USA, Canada, and China [12].

Ivory Coast experienced the demographic, economic growth, and strong urbanization (rate of urbanization = 50.3%) typical of many other Sub-Saharan African countries. Despite this growth, an epidemiological transition of non-communicable disease is underway, and the rise and public health consequences of overweight and obesity are evident. In 2005, the national nutrition reference study revealed that the prevalence rates of overweight and obesity among adults were 39.5% and 18.4%, respectively [13]. Moreover, non-communicable diseases estimated by World Health Organization (WHO) in 2008 accounted for 31% of total deaths [14]. However, data on obesity in Ivory Coast are scarce and old, and we lack a good description of the entire population as the data are focused on a small part of the population living mainly in Abidjan. In the earliest study, realized in 2015, Abidjan showed a prevalence of overweight at 43% among women with 13% of obesity [15]. Furthermore, the prevention and control of non-communicable disease are almost neglected. A comprehensive study is in great need to find countermeasures to control overweight and obesity. The objective of the current study was the epidemiologic profile of Ivorian adults affected by overweight and obesity. Specifically, we aimed to describe their sociodemographic characteristics, medical history, and lifestyle, analyze their clinical and biological parameters, to determine the prevalence of lipid disorders and related affection to overweight and obesity, and finally, to identify the potential risk factors of obesity.

## Methods

### Study design and sampling

We performed a cross-sectional study at the adult nutrition unit of the National Public Health Institute of Ivory Coast situated in Abidjan. Ivory Coast is situated on the west coast of Africa and bordered on the north by Mali and Burkina Faso, on the south by the Atlantic Ocean, on the west by Guinea and Liberia, and on the east by Ghana. Abidjan, with an estimated population of 4,395,243, is the largest city, and the economic, commercial, educational, and cultural center of the country. The adult nutrition unit is the unique public health reference center in the management of nutritional diseases in Ivory Coast. This study target population was people affected by obesity and overweight followed in this unit between January 2014 and July 2017. After addressing ethical considerations and obtaining authorization from the institution, we selected the new diagnosed patients during this period and using our inclusion criteria, namely: age above 17 years, available complete data in the folder and a complete biologic test (glycemia, total cholesterol, HDL cholesterol, triglyceride and uric acid). We got a sample of 2,643 patients among more than 6,500 patients.

Specific data included demographic characteristics (sex, age, occupational status, parity), medical and family history (existence of previous nutritional disorders and previous medical disease, existence of family disorders such as diabetes and obesity or overweight), lifestyle habits (physical activity, consumption of tobacco and alcohol), clinical parameters (weight, height, waist circumference), and biological parameters (glycemia, total cholesterol, HDL cholesterol, LDL cholesterol, triglycerides, uric acid).

### Anthropometric measurements

Concerning anthropometric measurements, the weight was obtained with the participant in the standing position, wearing light clothing and no footwear, using a TANITA Digital Weighing Scale HD 319 (TANITA Corp., Tokyo, Japan) to the nearest 0.1 cm and 0.1 kg. The stature (height) of bare-footed respondents was measured using the SECA Portable Stadiometer 213 (SECA GmbH & Co.KG, Hamburg, Germany). The average value of two measurements was used for data entry to minimize the measurement error. Following World Health Organization recommendations, body mass index (BMI) was calculated by dividing weight (kg) by the square of the height (m²), waist circumference was measured in centimeters using an inelastic tape measure placed directly on the skin at the level of the half distance between the last rib and the iliac crest, and blood was tested after at least eight hours of fasting in the morning with enzymatic methods on biochemical automate name COBAS Integra 400.

### Statistical analyses

The data analysis was performed on SPSS 20.0. Descriptive statistics were used to illustrate the sociodemographic and other characteristics of the respondents according to the BMI status. Pearson’s Chi-square test for categorical variables was used to analyze differences in characteristics between the study variables and BMI status. Binary logistic regression analyses were conducted at the multivariate levels using a backward stepwise method to explore the joint effects of the independent variables on the dependent variable. Binary logistic regression was used because it is the main statistical tool used to estimate associations between dependent and independent variables when dependent variables are constructed as a binary outcome. Significance was set at p = 0.05. The dependent variables were body mass index (BMI) and waist circumference (WC), and the independent variables were characterized by background characteristics. Where necessary, some of the independent variables were transformed or re-coded in order to make the analysis manageable and to facilitate comparison across the surveys.

We considered five occupational status (not working, professional/managerial, sales/trade, agricultural, and manual labor), five major ethnic groups (Akan, North Mande, South Mande, Gur, and Voltaique), parity into 2 categories (nulliparous and parous), physical activity into sufficient or not based on 150 minutes of moderate or vigorous activity per week. Furthermore, BMI data were divided into overweight (BMI of 25.0–29.9 kg/m^2^), and obesity type I (BMI of 30.0–34.99 kg/m^2^), type II (BMI of 35.0–39.99 kg/m^2^), and type III (BMI of BMI ≥ 40.0 kg/m^2^). Abdominal obesity was defined using waist circumference equal to or above 102 cm for men and 88 cm for women. Dyslipidemia was defined as hypercholesterolemia (total cholesterol >2.0 g/l), high LDL cholesterol (>1.3 g/l), low HDL cholesterol (<0.4 g/l), high triglyceride (serum triglyceride >1.5 g/l), and High TC/HDL-c (>5 for men and >4 for women).

## Results

### 1. Sociodemographic characteristics, medical history, and lifestyle of people affected by overweight and obesity

The majority of our respondents were females (86.3%) living in Abidjan (85.7%), who were at least 35 years old (78.5%) with an average age of 43.7 years (Table 1). Patients carrying out managerial activities were most represented (49.6%) followed by traders (25.2%). More than half belonged to the Akan ethnic group (51.6%). Regarding parity, we noticed that nearly two in three women had already gave birth (66.3%).

**Table 1:** Sociodemographic characteristics, medical history, and lifestyle of people affected by overweight and obesity.

Demographic characteristics, medical and family history		Number	Percentage (%)

Gender	Female	2,282	86.3
	Male	361	13.7
Age	≤24	115	4.4
	25–34	451	17.1
	35–44	867	32.8
	45–54	733	27.7
	≥55	477	18
Occupation	Agricultural and manual labour	77	2.9
	Not working	588	22.2
	Professional/managerial	1,311	49.6
	Sales/trade	667	25.2
Ethnic [Ivoirian = 2410 (91.2%)]	Akan	1,244	51.6
	North Mande	443	18.4
	Krou	351	14.6
	Voltaïque	308	12.8
	South Mande	64	2.7
Parity (Female = 2282)	Never give birth	519	22.7
	Already give birth	1,763	66.3
Residence	Abidjan	2,265	85.7
	Other	378	14.3
Previous nutritional disorders	Yes	1,295	49
	No	1,348	51
Type of nutritional disorders (n = 1295)	Overeating	1,112	85.9
	Nibble	183	14.1
Family history of overweight/obesity	Yes	599	22.7
	No	2,044	71.3
Alcohol consumption	Yes	883	33.4
	No	1,760	66.6
Tobacco smoking	Yes	180	6.8
	No	2,463	93.2
Sport practice	Yes	648	24.5
	No	1,995	75.5
Time of sport practice (n = 648)	<150 minutes	365	56.3
	≥150 minutes	283	43.7

Medical history and lifestyle focused on the factors that can induce obesity and are described as percentages. We found that 49% of the patients were affected by nutritional disorders with a clear preponderance of overeating (85.9%). A family history of overweight and obesity was found in 22.7% of patients. Alcohol consumption and tobacco smoking were found, respectively, in 33.4% and 6.8% of patients. Only 24.5% practiced sport and most of them (56.3%) were doing it for less than 150 minutes per week (Table 1).

### 2. Clinical and biological parameters of people affected by overweight and obesity

**Figure 1:**
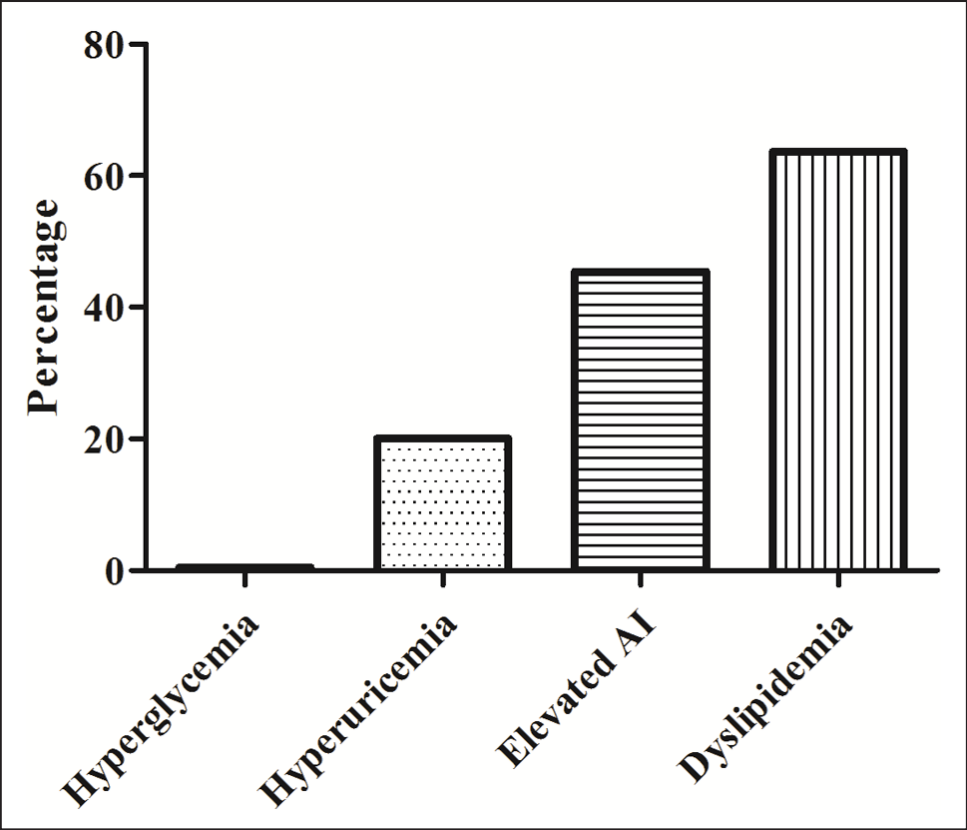
Biologic abnormalities of people affected by overweight and obesity in the Ivory Coast followed between 2014 and 2017 in the adult nutrition unit of INSP.

Most of the patients were affected by obesity (83.3%) with a clear preponderance of Obesity type I. Central obesity was found in 87.2% of the patients (Table 2). Concerning biological parameters, Figure 1 shows that 0.5% of the patients were affected by hyperglycemia, 20.1% by hyperuricemia, 45.4% by elevated atherogenicity index (AI), and 63.7% by dyslipidemia. Obesity complications were found in 78.5% of the patients and they were mainly metabolic. Osteo-articular and cardiovascular complications were found in 25% and 4% of patients, respectively (Table 3).

**Table 2:** Clinical and biological parameters of people affected by overweight and obesity.

Clinical parameters		Number	Percentage (%)

BMI	Overweight	441	16.7
	Obesity type I	1,008	38.1
	Obesity type II	729	27.6
	Obesity type III	465	17.6
Waist circumference	Normal	339	12.8
	High	2,304	87.2

**Table 3:** Medical complications among people affected by overweight and obesity in the Ivory Coast followed between 2014 and 2017 in the adult nutrition unit of INSP.

Obesity complications		Number	Percentage (%)

Obesity complications	Yes	2,076	78.5
	No	567	21.5
Type of affection	Metabolic	1,854	89.3
	Bones and joints	519	25
	Cardio-vascular	83	4
	Digestive	42	2
	Gyneco-obstetric	39	1.9
	Respiratory	4	0.2
	Social and cutaneous	4	0.2

### 3. Risk factors for overweight and obesity

At the bivariate level using a Chi-square test, a significant association has been found between gender, occupation, ethnicity, family history of obesity, physical activity, and obesity (Tables 4, 5). In multivariate analysis, the same factors remained significant with the specification of the impact of the length of physical activity. The most affected groups were females (p < 0.001), unemployed people (p < 0.001), salespersons and traders (p < 0.001), members of the North Mande ethnicity (p = 0.018), those with a family history of obesity (p < 0.001), and those whose practicing sport less than 150 minutes per week (p = 0.023; Table 6). Furthermore, we found a strong positive correlation between BMI and waist circumference (WC) (r = 0.73; p < 0.001).

**Table 4:** Bivariate analysis of sociodemographic characteristics, BMI, and waist circumference.

		BMI			Waist circumference		

		Overweight	Obesity	P	Normal	Abnormal	P

Age	≤24 years	19	96	0.640	23	92	0.014
		(16.5%)	(83.5%)		(20.0%)	(80.0%)	
	25–34 years	79	372		62	389	
		(17.5%)	(82.5%)		(13.7%)	(86.3%)	
	35–44 years	153	714		119	748	
		(17.6%)	(82.4%)		(13.7%)	(86.3%)	
	45–54 years	121	612		92	641	
		(16.5%)	(83.5%)		(12.6%)	(87.4%)	
	≥55 years	69	408		43	434	
		(14.5%)	(85.5%)		(9.0%)	(91.0%)	
Gender	Male	112	249	<0.001	150	211	0.003
		(31.0%)	(69.0%)		(41.6%)	(58.4%)	
	Female	329	1953		189	2093	
		(14.4%)	(85.6%)		(8.3%)	(91.7%)	
Parity	0	86	433	0.066	68	451	<0.001
		(16.6%)	(83.4%)		(13.1%)	(86.9%)	
	≥1	243	1520		121	1642	
		(13.8%)	(86.2%)		(6.9%)	(93.1%)	
Occupation	agricultural and manual labour	13	64	<0.001	8	69	<0.001
		(16.9%)	(83.1%)		(10.4%)	(89.6%)	
	Unemployed	71	517		58	530	
		(12.1%)	(87.9%)		(9.9%)	(90.1%)	
	professional/managerial	298	1013		236	1075	
		(22.7%)	(77.3%)		(18.0%)	(82.0%)	
	Salesperson/trader	59	608		37	630	
		(8.8%)	(91.2%)		(5.5%)	(94.5%)	
Ethnic	Akan	241	1003	0.010	181	1063	0.040
		(19.4%)	(80.6%)		(14.5%)	(85.5%)	
	Krou	64	287		55	296	
		(18.2%)	(81.8%)		(15.7%)	(84.3%)	
	North Mande	58	385		47	396	
		(13.1%)	(86.9%)		(10.6%)	(89.4%)	
	South Mande	7	57		10	54	
		(10.9%)	(89.1%)		(15.6%)	(84.4%)	
	Voltaïque	44	264		30	278	
		(14.3%)	(85.7%)		(9.7%)	(90.3%)	

**Table 5:** Bivariate analysis of medical history, lifestyle, BMI, and waist circumference.

Overweight		BMI			Waist circumference		

		Obesity	P	Normal	Abnormal	P	

Familial history of obesity	No	381	1663	<0.001	281	1763	0.009
		(18.6%)	(81.4%)		(13.7%)	(86.3%)	
	Yes	60	539		58	541	
		(10.0%)	(90.0%)		(9.7%)	(90.3%)	
Physical activity	No	302	1693	<0.001	218	1777	<0.001
		(15.1%)	(84.9%)		(10.9%)	(89.1%)	
	Yes	139	509		121	527	
		(21.5%)	(78.5%)		(18.7%)	(81.3%)	
Length of physical activity	<150 minutes	87	278	0.093	72	293	0.440
		(23.8%)	(76.2%)		(19.7%)	(80.3%)	
	≥150 minutes	52	231		49	234	
		(18.4%)	(81.6%)		(17.3%)	(82.7%)	
Nutritional disorders	No	228	1120	0.750	178	1170	0.550
		(16.9%)	(83.1%)		(13.2%)	(86.8%)	
	Yes	213	1082		161	1134	
		(16.4%)	(83.6%)		(12.4%)	(87.6%)	

**Table 6:** Binary logistic regression of BMI, sociodemographic parameters, medical history, lifestyle, and obesity among patients followed between 2014 and 2017 in the adult nutrition unit of INSP.

		Coefficient	P	OR

Sex	Male (reference)			
	Female	0.725	<0.001	2.064
Profession			<0.001	
	Manager (reference)			
	Agriculture	0.41	0.196	1.506
	Unemployed	0.598	<0.001	1.818
	Sales Person/trader	0.885	<0.001	2.423
Ethnic group			0.063	
	Akan (reference)			
	Krou	0.119	0.457	1.126
	Voltaique	0.271	0.139	1.311
	North Mande	0.384	0.018	1.469
	South Mande	0.791	0.057	2.206
	Foreigner	0.342	0.128	1.408
Family history of obesity	Yes (reference)			
	No	–0.671	<0.001	0.511
Sport practice ≥150 min	Yes	–0.323	0.023	0.724
	No (reference)			
Constant		1.153	<0.001	3.168

Abdominal obesity was significantly influenced by age, gender, parity, occupation, ethnicity, family history of obesity, and physical activity at the bivariate level (Tables 4, 5). In multivariate analysis, age, gender, parity, and obesity were significant and nutritional disorders appeared as a factor. As Table 7 shown, abdominal obesity was found mostly in people aged above 34 years, females (p < 0.001), women whose already gave birth (p = 0.012), obese patients (p < 0.001), and patients with nutritional disorders (p = 0.041).

**Table 7:** Binary logistic regression of WC, sociodemographic parameters, medical history, lifestyle, and obesity among patients followed between 2014 and 2017 in the adult nutrition unit of INSP.

		Coefficient	P	OR

Age			0.002	
	<25 years (reference)			
	25–34 years	0.52	0.107	1.682
	35–44 years	0.664	0.032	1.943
	45–54 years	0.933	0.004	2.543
	≥55 years	1.31	<0.001	3.707
Sex	Male (reference)			
	Female	1.847	<0.001	6.342
Parity	Never gave birth (reference)			
	Already gave birth	0.488	0.012	1.629
Sport practice	No (reference)			
	Yes	–0.295	0.052	0.744
Nutritional disorder	No	–0.294	0.041	0.745
	Yes (reference)			
Clinical obesity	No (reference)			
	Yes	2.67	<0.001	14.445
Constant		–2.102	<0.001	0.122

## Discussion

Concerning sociodemographic characteristics, most of our patients were living in the city of Abidjan. This result could be explained by the fact that INSP is situated in Abidjan, but it is important to notice the rapid trend of obesity in urban cities due to the increase in the obesogenic environment characterized by changes in food supply systems, changes in diets (rich in saturated fatty acid diets, low in polyunsaturated fatty acids, and fibers) over traditional ones, and the declining levels of physical activity [6,16,17]. Like many cities in developing countries, Abidjan experienced a rapid urbanization, estimated at 50.3% in 2014 [18].

The majority of our patients were at least 35 years old; the average age was 43.7 years old, and the median was 43 years old. Pessinaba S in Senegal confirmed the higher prevalence of obesity in patients above 43 years old [19]. Vigy also found an average age of 43.5 years among her obese patients.

Females were the dominant gender, and they were two times more affected by obesity than men according to a multivariate analysis. The preponderance of obesity in women has been confirmed in many studies in Sub Sahara Africa and in other parts of the world [20,21]. Women obesity, particularly in Sub Sahara Africa, could be explained by the social desirability for overweight and obese women. In many ethnicities in this part of the world and in Ivory Coast, large-sized women are perceived as beautiful and prestigious, so overweight and obese women are valorized, admired, and respected by society, and prized by their husbands and families [22]. Also, overweight gynoids and obese women are symbols of fertility and sensuousness, and most of the men find them sexually attractive [23]. Thus, many ethnicities encourage female obesity by overfeeding. This habit is more accentuated among the north Mande ethnicity and could explain the fact that in our study, this group was 1.5 times more affected than the other ethnicities. In Mali, Diopi et al. confirmed the higher prevalence of obesity in North Mande [24].

Concerning occupation, in bi- and multivariate analysis, unemployed and salespersons were the most affected. The explanation for this observation could be the low level of physical activity among this population. The same result has been found in Ghana by Tuoyire et al. In their study, sales/trade occupations were more affected by overweight and obesity [25]. Erem et al. also found a high prevalence of obesity among unemployed and salespersons [26]. Furthermore, salespersons were slightly more affected than unemployed in our study. This could be explained by the quality of food eaten by salespersons. In fact, because of their activities, most of them are used to eat fast food sold on the market, especially a semolina of cassava fermented and steamed named “attiéké”which is rich in carbohydrates and the most popular meal consumed in Ivory Coast, while unemployed people have opportunities to eat food of better quality [27]. Interestingly, Gbakayoro et al. showed that attiéké caused high post-prandial glycemia and is responsible for a rapid increase in the BMI and abdominal obesity. Moreover, these foods are mostly eaten accompanying with fried fish or a fat sauce, like peanut sauce or palm oil sauce, which increases the ingestion of calories.

A family history of obesity was found in our study at 22.7%, a percentage that is lower than those found by Gallissot-pierrot (55.8%) and Hajian-tilaki et al. (35.3%)[28]. We also found that people with a family history of obesity were two times more affected by obesity. Erem et al. and Wang et al. reported a similar result in their studies [26,29].

Concerning lifestyle, only 13.8% of the patients were practicing physical activity for at least 150 minutes weekly. These data are confirmed by the nutritional reference study conducted in 2005, which revealed a low level of physical activity (<600 MET-minutes/week) in 88.1% of the adult population. This attitude is due to the decrease of physical activity with age [30]. Moreover, physical inactivity was significantly associated with obesity in bi- and multivariate analysis as described in many studies [31,32]. Patients with a 150 minutes weekly practice of sports were less affected by obesity. Little et al. showed that each hour of moderate physical activity was associated with a 0.085 kg/m^2^ decrease in BMI, and higher physical activity was associated with decreased odds of obesity class I and obesity class II. These data confirm the health benefit that may confer a modest level of physical activity. Besides physical inactivity, we have noticed the presence of nutritional disorder at 49% among our patients with a clear preponderance of overeating. Vigy and Gallissot-Pierrot found nutritional disorders in, respectively, 58% and 45.3% of their obese patients; in the study by Gallissot-Pierrot, nutritional disorders were dominated by Nibble.

Clinically, we observed abdominal obesity in 87.2% of the patients, and a strong correlation between abdominal obesity and BMI was found. Hu et al. found a similar result among his obese participants, with the prevalence of central obesity estimated at 63.3% and a correlation between BMI and WC estimated at 0.721 [33]. Furthermore, we have seen that the prevalence of central obesity is increasing with age, mainly in those 34 years old or older. This increasing prevalence of central obesity with age has been shown by Erem et al. Concerning gender, females were 6.3 times more affected than males. Hu et al. found the same result [33]. Maimela et al., Wang et al., and Erem et al. showed a significantly high prevalence among their women population, respectively at 49.8%, 43.9% and 38.9% [26,29,34]. This variability in the prevalence of central obesity between men and women might be explained by the difference in lifestyle, sociodemographic parameters, and genetic characteristics, including parity, which were found as an influencing factor. Concerning nutritional disorder, we noticed that people without nutritional disorders were less affected. The influence of nutritional disorders or diet has been found by Reynolds et al. in their study in which diet explained 44.8% and 48.3% of the excess risk of central obesity among men and women, respectively, in urban areas of China [35].

At the level of complications, the majority of our patients were affected by metabolic complications characterized mainly by dyslipidemia. Doupa et al. in Senegal found a prevalence of dyslipidemia at 63.8% among their population dominated by obese people [31]. In Ivory Coast, Lokrou et al. revealed a prevalence of dyslipidemia at 47.4% among the diabetic patients most affected by obesity [36].

There are some limitations to this study. First, the study was a cross-sectional study from which we cannot infer causal relationships between dependent and independent variables. Second, some recall bias could happen. Third, the numbers of females were very high and could potentially induce gender bias. Nevertheless, as we are waiting for prospective studies, these results could help fighting overweight and obesity in Ivory Coast.

## Conclusions

In summary, the cross-sectional study shows that females, older age and occupation were associated with greater affection risks of overweight and obesity in patients. Ethnic group, pregnancy and family history of obesity posed a lower but significant risk for obesity. More sport practice played a protective role against the acquisition of overweight and obesity. The prevalence of dyslipidemia among overweight or obese people was high. This founding provides a clue for further studies on the obesity and health in the population of that region.
